# The Cambridge Course

**Published:** 1908-09-05

**Authors:** 


### THE CAMBRIDGE UNIVERSITY COURSE.

The degrees of Bachelor of Medicine (M.B.) and Bachelor of Surgery (B.C.) at this University are only conferred after the entire course of study with examinations for both has been carried out. The B.C. is, therefore, in itself a complete and registrable qualification to practise, and is frequently so used. To obtain either of these degrees it is necessary to pass five years in the study of medicine after registration as a student, and to reside for three years at the University. It is not necessary to take any degree or examination in arts, except the Previous examination, which is a compulsory preliminary to every degree course, but most medical students at Cambridge take the B.A. degree in a Tripos or Pass examination.

The Previous examination should be passed at the commencement of the first term of residence, unless exemption has already been obtained. The professional examinations are three in number The first comprises physics, chemistry, and biology; like similar examinations elsewhere, it is divided into two parts, which may be passed separately. Those who have already studied the subjects before coming into residence and have passed the Previous examination, will find little difficulty in passing both parts at the end of their first term; for others, a moderate diligence should produce a similar reward at the end of a year. The second absorbs at least two years' work, and frequently more; the standard in anatomy and physiology, which must be passed together, is very high, and although the teaching of these subjects in the University is unsurpassed, the percentage of failures in this examination is always large. During the period of preparation for this examination, the medical student who is desirous of an Arts degree must also arrange his studies to that end. In the third, or Final examination, there are two parts : one consists of pathology, bacteriology, pharmacology, etc., and may be taken as soon as various courses of lectures and laboratory work in those subjects have been duly attended; the other includes medicine, surgery, and midwifery with gynæcology, and it may not be attempted until the completion of the scheme of medical study, which includes three years' attendance on the practice of a recognised hospital.

In pathology and the other subjects of the first part of the Final examination there are excellent courses of lectures and demonstrations in the University laboratories, and Long Vacation courses in these subjects are especially popular. For the remaining part it is usual to study at one of the large London or provincial medical schools, though there are facilities in the University and at Addenbrooke's Hospital for completing the entire course without leaving Cambridge. This examination is searching, but eminently practical in character; the examiners devote themselves to finding out what a man knows of his work, rather than to tripping him on catch points of no intrinsic importance. Many students who present themselves for it have already obtained a registrable qualification elsewhere and fulfilled a resident hospital appointment; such rarely fail at Cambridge. At the same time an unqualified man has an excellent chance if he has made the most of his opportunities in the wards and out-patient rooms of a large hospital.

When the third examination has been completed the student may take the degree of B.C. at once, but for the M.B. he must prepare and submit a thesis on some subject in medicine, surgery, or midwifery, selected by himself. Although original research is not essential, this thesis must bear evidence that it is founded on the personal observations and reflections of the author.

For the degree of M.D. it is necessary to have been M.B. at least three years, and to submit a thesis on some subject in medicine (not in surgery), which is a definite research or contribution to the knowledge of its subject. A degree of Master in Surgery (M.C.) is also given by the University, as well as diplomas in public health and tropical medicine.

